# Repurposing anti-diabetic drug “Sitagliptin” as a novel virulence attenuating agent in *Serratia marcescens*

**DOI:** 10.1371/journal.pone.0231625

**Published:** 2020-04-16

**Authors:** Hisham A. Abbas, Wael A. H. Hegazy

**Affiliations:** Department of Microbiology and Immunology, Faculty of Pharmacy, Zagazig University, Zagazig, Egypt; University of Toledo Health Sciences Campus, UNITED STATES

## Abstract

**Background:**

*Serratia marcescens* is an emerging pathogen that causes a variety of health care associated infections. *S*. *marcescens* is equipped with an arsenal of virulence factors such as biofilm formation, swimming and swarming motilities, prodigiosin, protease and others which enable it to initiate and cause the infection. These virulence factors are orchestrated under the umbrella of an intercellular communication system named Quorum sensing (QS). QS allows bacterial population to synchronize the expression of virulence genes upon detection of a chemical signaling molecule. Targeting bacterial virulence is a promising approach to attenuate bacteria and enhances the ability of immune system to eradicate the bacterial infection. Drug repurposing is an advantageous strategy that confers new applications for drugs outside the scope of their original medical use. This promising strategy offers the use of safe approved compounds, which potentially lowers the costs and shortens the time than that needed for development of new drugs. Sitagliptin is dipeptidyl peptidase-4 (DPP-4) inhibitor, is used to treat diabetes mellitus type II as it increases the production of insulin and decreasing the production of glucagon by the pancreas. We aimed in this study to repurpose sitagliptin, investigating the anti-virulence activities of sitagliptin on *S*. *marcescens*.

**Methods:**

The effect of sub-inhibitory concentrations of sitagliptin on virulence factors; protease, prodigiosin, biofilm formation, swimming and swarming motilities was estimated phenotypically. The qRT-PCR was used to show the effect of sitagliptin on the expression of QS-regulated virulence genes. The *in-vivo* protective activity of sitagliptin on *S*. *marcescens* pathogenesis was evaluated on mice.

**Results:**

Sitagliptin (1 mg/ml) significantly reduced the biofilm formation, swimming and swarming motilities, prodigiosin and protease. The qRT-PCR confirmed the effect on virulence as shown by down regulating the expression of *fimA*, *fimC*, *flhC*, *flhD*, *bsmB*, *rssB*, *rsmA*, *pigP*, and *shlA* genes. Moreover, the *in-vivo* findings showed the efficient ability of sitagliptin to weaken *S*. *marcescens* pathogenesis.

**Conclusion:**

Sitagliptin is a promising anti-virulence agent against *S*. *marcescens* that may be beneficial in the control of healthcare associated infections caused by *S*. *marcescens*.

## Introduction

*Serratia marcescens* is a facultative anaerobic motile Gram negative rod that is considered a member of the family Enterobacteriaceae. *S*. *marcescens* was considered a saprophytic nonpathogenic organism, until its first known outbreak of nosocomial infection in 1951. Since then, nosocomial infections caused by this organism have been reported frequently [1,2]. *S*. *marcescens* was reported the seventh most frequent pathogen that is responsible for nosocomial pneumonia and the tenth most frequent one that causes for hospital acquired blood stream infections [3]. Being ubiquitous inhabitants of soil, water, animals, insects, plant and surfaces, *S*. *marcescens* is an opportunistic human pathogen that can cause a variety of nosocomial infections [2]. *S*. *marcescens* is one of the opportunistic food-borne pathogens, due to its capability to colonize wide variety of gastrointestinal tract surfaces. It is one of the important nosocomial pathogens that causes intravenous catheter-associated infections, pneumonia, endocarditis, urinary tract infections and osteomyelitis [2,4]. The pathogenesis of *S*. *marcescens* is attributed to swimming and swarming motilities in addition to its extracellular enzyme activities (e.g., protease, nuclease, hemolysin and lipase) [2]. Moreover, the resistance of *S*. *marcescens* to many antibiotics as β-lactam, aminoglycosides and fluoroquinolones exaggerated its pathogenesis [5,6].

It has been discovered that the bacterial population is influenced by cell–cell communication via small molecules produced and released by bacterial cell as ‘words’ which reach other bacterial cell to elicit ‘answers’. The chemically encrypted language that used to organize a uniformed expression of virulence genes is called quorum sensing (QS) [7]. The quorum sensing signaling system controls diverse physiological functions in *S*. *marcescens*; examples are swarming motility, haemolytic activity, production of biofilm [8], sliding motility, production of biosurfactant, prodigiosin, nuclease [9,10], production of antibiotics [10], production of enzymes as nuclease, chitinase, protease, lipases, antibacterial compound and butanediol fermentation [11]. It is understood now that, QS regulates the expression of numerous genes associated with virulence factor production and biofilm formation; which may augment antibiotic resistance [12,13]. As a consequence, the treatment of some of *S*. *marcescens* resistant strains is difficult; especially with the increased resistance to several antibiotic classes such as β-lactam, aminoglycosides and quinolones [5,6].

Repurposing of ‘old’ drugs is a strategy for identifying new uses for approved drugs other than their original medical uses. Drug repurposing is increasingly becoming an attractive proposition, as it offers various advantages. Importantly, the repurposed drug’s safety has already been pre-approved and the formulation development already has been completed. This strategy lowers the coasts and shortens the time that needed to develop new drug [14]. Attenuation of bacteria is one of the alternative strategies to overcome bacterial pathogenesis via targeting its QS [15,16]. In this direction, several working groups studied the capability of some compounds as anti-biofilm and anti-QS agents [13,17]. Among these compounds, nitrogenous heterocyclic compounds such as pyrazine dicarboxylic acid derivatives were potentially used to modulate the QS of *Vibrio cholera*; they simply targeted the global response regulator LuxO. Pyrazine derivatives showed anti-biofilm activity and reduced the adhesion and invasion of the Vibrios onto the intestinal cell lines [18]. Triazoles are nitrogenous heterocyclic moieties that were abundantly used as antibacterial drugs such as tazobactam and cefatrizine [19]. Many triazole moiety-containing compounds have the capability to bind various biological targets via hydrogen bonding and dipole interactions [19,20]. Interestingly, triazole derivatives showed anti-QS activity, especially those containing analogs of natural N-acyl L-homoserine lactone, thymidine and isoxazole structures that strongly modulated bacterial QS and can be used as potential lead structures for the development of effective QS inhibitors [21–23]. In our previous work, diverse compounds which can modulate QS to curtail bacterial pathogenesis were screened. Bearing this in mind, our study aimed to investigate the ability of sitagliptin as a drug that is pyrazine derivative with triazole moiety to serve as an inhibitor of *S*. *marcescens* virulence *in-vitro* and *in-vivo*.

## Materials and methods

### Media and chemicals

Mueller Hinton (MH) broth, Mueller Hinton (MH) agar and Tryptone soy broth (TSB) were the products of Oxoid (Hampshire, UK). Luria-Bertani (LB) agar and LB broth were purchased from Lab M Limited (Lancashire, United Kingdom). Sitagliptin was obtained from Sigma-Aldrich (St. Louis, USA). Other used chemicals were of pharmaceutical grade.

### Bacterial strains

The *S*. *marcescens* isolate in this study is a clinical one obtained from an Intensive Care Unit patient admitted to Zagazig University Hospital by endotracheal aspiration [24]. The bacterial isolate was not collected specifically for this study, it was collected from admitted patient in the Zagzig Univesity Hospital’s Intensive Care Unit, and the patient consent was obtained for microbiological and pathological examination (according to the routine protocols used in hospital for admitted patients) by hospital administration department in complete comply to Helsinki declarations without any burden, risk or danger on the patient. The Matrix-Assisted Laser Desorption/Ionization-Time Of Flight (MALDI-TOF) mass spectrometry instrument at the Clinical Pathology Department, Faculty of Medicine, Zagazig University was used for identification of this isolate. The identification was based on ribosomal proteins and the identity percentage was 100%.

### Determination of minimum inhibitory concentration (MIC)

The agar dilution method was used in determination of the minimum inhibitory concentration of sitagliptin according to the Clinical Laboratory and Standards Institute Guidelines (CLSI, 2012). The tested strain was incubated overnight in TSB and the suspension was diluted with MH broth in order to prepare a suspension with a turbidity approximating that of 0.5 McFarland Standard. The suspension was further diluted with sterile saline (1:10). By using a micropipette, a standardized inoculum (approximately 10^4^ CFU per spot) was spotted on the surface of MH agar plates containing different sitagliptin concentrations and control plate without sitagliptin. The MIC of sitagliptin was the lowest concentration that inhibits growth on the plate after incubation at 37°C for 20 hr.

### Effect of sitagliptin on bacterial growth

The effect of sub-inhibitory concentration of sitagliptin on the growth of the tested strain of *S*. *marcescens* was detected according to Nalca *et al*. [25]. Overnight culture from *S*. *marcescens* was prepared in LB broth and adjusted to 0.5 McFarland Standard. The prepared suspension was used to inoculate LB broth containing 1 mg/ml of sitagliptin and control LB broth without sitagliptin so that the final inoculum is approximately 1×10^8^ CFU/ml. After overnight incubation at 37°C, the optical densities of both cultures were measured at 600 nm by using Biotek Spectrofluorimeter (Biotek, USA). The experiment was performed in triplicate and data are presented as the mean ± standard error. A *P* value < 0.05 was considered statistically significant using Student's t-test with (Graphpad Prism 5 software).

Sub-inhibitory concentration of sitagliptin was used to investigate its anti-virulence and anti-quorum sensing activities on *S*. *marcescens*. The reason for the use of this concentration is to avoid any effect on the growth of the tested bacterial strain. The OD_600_ of sitagliptin sub-MIC-treated (1 mg/ml) and untreated cultures of *S*. *marcescens* were compared to show that the growth was not affected by sitagliptin treatment. For normalizing the results in all the next experiments, the sitagliptin treated or untreated bacterial cultures were adjusted to the growth density OD_600_ of 0.4 (1×10^8^ CFU/ ml).

### Biofilm inhibition assay

The tested strain was reported as a strong biofilm forming isolate [24]. To determine the ability of sitagliptin to inhibit biofilm formation, the modified method of Abraham *et al*. was used [26]. A suspension of *S*. *marcescens* strain was prepared from overnight culture in TSB and its optical density was adjusted to OD_600_ of 0.4 (1×10^8^ CFU/ ml) was added. Aliquots of 10μl of the suspension were added to 1 ml amounts of fresh TSB with and without 1 mg/ml of sitagliptin. Aliquots of 100 μl of TSB with and without sitagliptin were delivered into the wells of 96 wells microtiter plate and incubated at 28°C for 24 hr. The planktonic cells were aspirated and the wells were washed three times with distilled water and left to dry. The attached cells were fixed with methanol for 20 min and stained with crystal violet (1%) for 20 min. The wells were washed and the attached dye was eluted by 33% glacial acetic acid. The absorbance was measured at 590 nm using Biotek Spectrofluorimeter (Biotek, USA). The experiment was repeated triplicate and the results were averaged. The absorbance of sitagliptin treated *S*. *marcescens* were expressed as mean ± standard error of percentage change from untreated *S*. *marcescens* control. The percentage of biofilm inhibition was calculated using the following formula:

$$[OD_{590}control‐OD_{590}\;in\;presence\;of\;sitagliptin]/OD_{590}\;control$$

### Microscopic visualization of biofilm inhibition by the light microscope

In order to analyze biofilm inhibition, the method of Sakar *et al*. [27] was followed with some modification. The biofilm of the tested strain of *S*. *marcescens* was formed on glass slides placed in polystyrene petri plates in the presence and absence of 1 mg/ml of sitagliptin. The plates were incubated for 24 hr at 28°C; the slides were washed with water three times and stained with crystal violet (1%) for 20 min. The slides were examined after staining under the light microscope at a 100X magnification.

### Swimming and swarming motilities assay

The ability of sitagliptin to block the swimming and swarming motilities was detected according to Matsuyama *et al*. [28]. For swimming assay, LB agar plates with 0.3% agar with and without 1 mg/ml sitagliptin were prepared. Overnight culture of *S*. *marcescens* in LB broth (OD_600_of 0.4) was prepared and 5μl of the suspension was inoculated into the center of the plates. Swarming LB gar plates with 0.5% agar containing 1 mg/ml of sitagliptin and control plates were point inoculated with 5μl of the prepared suspension. The plates were incubated at 28°C for 20 hr. The zones of swimming or swarming were measured and the experiment was done in triplicates and the obtained results were averaged.

### Prodigiosin inhibition assay

The production of prodigiosin by *S*. *marcescens* was quantified in the presence and absence of sitagliptin. The optical density of *S*. *marcescens* suspension was adjusted to OD_600_ of 0.4 (1×10^8^ CFU/ ml) and inoculated in 2 ml fresh LB broth with or without sitagliptin sub-MIC at 28°C for 18 hr. The cells were collected by centrifugation at 13000 rpm for 10 min. To extract prodigiosin, acidified ethanol (4% 1M HCl in ethanol) was used. The absorbance was measured at 534 nm using Biotek Spectrofluorimeter (Biotek, USA) and the degree of inhibition was determined. The experiment was made in triplicate and the results were averaged [29]. The absorbance of sitagliptin treated *S*. *marcescens* cultures were expressed as mean ± standard error of percentage change from untreated *S*. *marcescens* control. The percentage of prodigiosin inhibition was calculated using the following formula:

$$[A_{534}control‐A_{534}\;in\;presence\;of\;sitagliptin]/A_{534}\;control$$

### Protease assay

In order to determine the protease inhibitory activity of sitagliptin, the skim milk agar method was used [30]. *S*. *marcescens* treated with sitagliptin sub-MIC or untreated overnight cultures in LB broth were adjusted to OD_600_ of 0.4, centrifuged at 10,000 rpm for 15 min and the protease activities were measured by adding the supernatants in 100 μl aliquots to the wells made in skim milk agar plates (5%). The plates were incubated overnight at 37°C and the diameters of the clear zones surrounding the growth were measured. The experiment was made in triplicate and the clear zones obtained by protease produced by sitagliptin treated *S*. *marcescens* cultures were expressed as mean ± standard error of percentage change from the protease inducing clear zones obtained by untreated *S*. *marcescens* control on skim milk agar plates. The percentage of protease inhibition was calculated using the following formula:

[Clearzonediameterofcontrol‐Clearzonediameterinpresenceofsitagliptin]/Clearzonediameterofcontrol

### Quantitative real-time PCR (qPCR) analysis

#### RNA extraction

Sitagliptin treated and untreated *S*. *marcescens* cultures (OD_600_ 0.4) were collected by centrifugation (6,000 rpm for 10 min, 4°C). Bacterial pellets were re-suspended in Tris-EDTA buffer (100 μL) provided with lysozyme and incubated for 5 min at 25°C. Bacterial pellets were lysed by RNA lysis buffers and total RNA was isolated and purified using RNAeasy Mini Kit (Qiagen, Germany) according to manufacturer instructions. DNase was used to remove any residual chromosomal DNA. Finally, RNA concentrations were measured by NanoDrop ND-1000 spectrophotometer and stored at -70°C until use.

#### Real time PCR (qRT-PCR)

The influence of sub-MIC of sitagliptin on expression levels of genes that encode and regulate bacterial adhesion *fimA*, *fimC* and *bsmB* [31], genes responsible for swarming and swimming *flhC*, *flhD*, *rssB* and *rsmA* [32,33], prodigiosin encoding gene *pigB* and pore-forming toxin encoding gene *shlA* [33]; was characterized using RT-qPCR. Total RNA (10 ng), from each sample; untreated *S*. *marcescens* and sitagliptin sub-MIC treated *S*. *marcescens*, was used for cDNA synthesis by reverse transcription using high capacity cDNA Reverse Transcriptase kit (Applied Biosystem, USA). The cDNA was subsequently amplified with the Syber Green I PCR Master Kit (Fermentas) in a 48-well plate using the Step One instrument (Applied Biosystem, USA) as follows: 10 minutes at 95ºC for enzyme activation followed by 40 cycles of 15 seconds at 95ºC, 20 seconds at 55–65 ºC and 30 second at 72 ºC for the amplification step. Changes in the expression of each target gene were normalized relative to the mean critical threshold (CT) values of *rplU* as housekeeping gene by the 2^*-ΔΔCt*^ method [31,33]. One μM of both primers specific for each target gene were used. Primers sequence and annealing temperature specific for each gene demonstrated in Table 1. The experiment was made in triplicate and the genes’ expression of sitagliptin treated *S*. *marcescens* were presented as mean ± standard error of fold change from untreated *S*. *marcescens* control.

**Table 1:** Sequences of the used primers and annealing temperatures for tested genes.

Target gene	Sequence (5’–3’)	Annealing Temp	Reference
***fimA***	**For:** ACTACACCCTGCGTTTCGAC	**58°C**	**[31]**
	**Rev:** GCGTTAGAGTTTGCCTGACC		
***fimC***	**For:** AAGATCGCACCGTACAAACC	**55**°C	**[31]**
	**Rev:** TTTGCACCGCATAGTTCAAG		
***flhc***	**For:** AAGAAGCCAAGGACATTCAG	**60°C**	**[33]**
	**Rev:** TTCCCAGGTCATAAACCAGT		
***flhD***	**For:** TGTCGGGATGGGGAATATGG	**55°C**	**[31]**
	**Rev:** CGATAGCTCTTGCAGTAAATGG		
***bsmB***	**For:**:CCGCCTGCAAGAAAGAACTT	**62°C**	**[33]**
	**Rev:** AGAGATCGACGGTCAGTTCC		
***rssB***	**For:**TAACGAACTGCTGATGCTGT	**58**°C	**[33]**
	**Rev:** GATCTTGCGCCGTAAATTAT		
***rsmA***	**For:** TTGGTGAAACCCTCATGATT	**65°C**	**[33]**
	**Rev:** GCTTCGGAATCAGTAAGTCG		
***pigP***	**For:** GAACATGTTGGCAATGAAAA	**55°C**	**[33]**
	**Rev:** ATGTAACCCAGGAATTGCAC		
***shlA***	**For:** GCGGCGATAACTATCAAAAT	**55°C**	**[33]**
	**Rev:** ATTGCCAGGAGTAGAACCAG		
***rplU***	**For:** GCTTGGAAAAGCTGGACATC	**65°C**	**[31]**
	**Rev:** TACGGTGGTGTTTACGACGA		

### Mice survival test

The protective activity of sitagliptin on *S*. *marcescens* pathogenesis was evaluated by the mice survival *in-vivo* model following the method of Kim *et al*. [34]. An approximate cell density of 1 x 10^8^ CFU/ml in phosphate-buffered saline (PBS) of *S*. *marcescens* was prepared from overnight bacterial cultures in LB broth with and without sub-MIC of sitagliptin (1mg/ml) and also in LB broth with DMSO in the same concentration that was used as a solvent for sitagliptin. Five random groups of three-weeks-old healthy female albino mice (Mus musculus) with the same weight were used, each comprising 5 mice. In Group 1, mice were injected intraperitoneally with 100 μl of sitagliptin-treated bacteria in sterile PBS; group 2 was injected with 100 μl of DMSO-treated bacteria, while group 3 was injected with 100 μl of untreated bacteria. Two negative control groups are included also; group 4 mice are injected with 100 μl of sterile PBS and group 5 mice were left un-inoculated. All mice groups were housed in plastic cages with wood shave bedding in the animal house of the Faculty of Pharmacy, Zagazig University, Egypt. The experimental animals were kept with normal aeration and feeding, under humidity (60±10%), controlled room temperature (25±2 ^o^C) and 12 hr light/dark cycle. The survival of mice in each group was recorded every day for 5 successive days. Mice survival in each group was recorded every day over 5-days period, plotted using Kaplan-Meier method and significance (*P* < 0.05) was calculated using Log-rank test, GraphPad Prism 5. After the end of experiment, only mice suffered from pathological conditions and/or loss of appetite and weight were anesthetized by thiopental and euthanized by cervical dislocation. All animal experiments have been approved by the Institutional Review Board (ethical committee) at the Faculty of Pharmacy, Zagazig University, which comply with the ARRIVE guidelines and carried out in accordance with the U.K. Animals (Scientific Procedures) Act, 1986 and associated guidelines.

## Results

### Determination of MIC

Sitagliptin inhibited the growth of *S*. *marcescens* isolate at 10 mg/ml. The concentration selected to test the anti-QS and anti-virulence activities of sitagliptin is 1 mg/ml which is equivalent to 1/10 MIC.

### Determination of the effect of sitagliptin on bacterial growth

**Fig 1:**
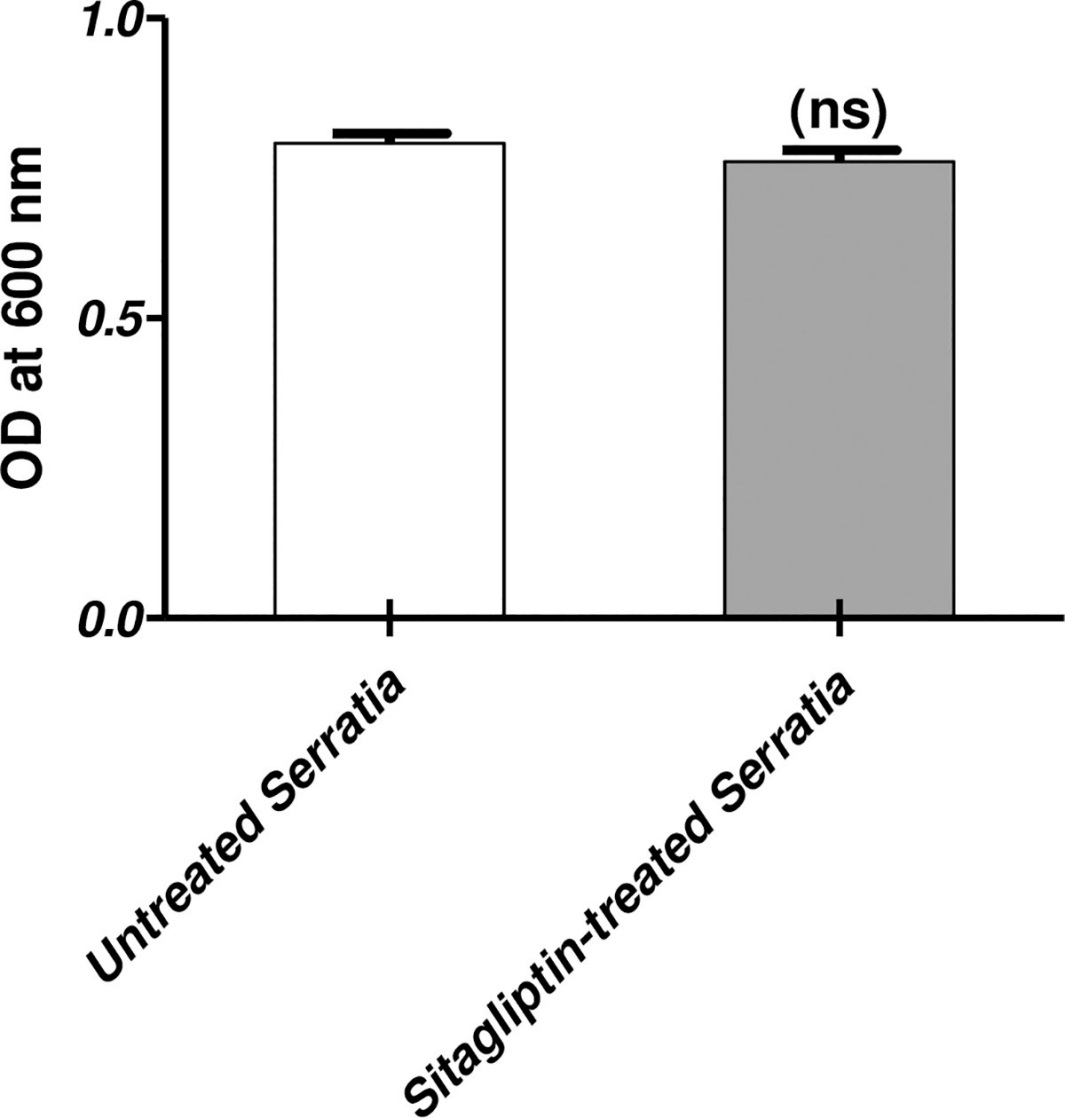
Effect of sitagliptin (sub MIC) on growth of *S*. *marcescens*. LB broth media containing 1 mg/ml of sitagliptin and control LB broth without sitagliptin were inoculated with an overnight culture from *S*. *marcescens* adjusted 0.5 McFarland Standard. After overnight incubation at 37°C, the optical densities of both cultures were measured at 600 nm. The Student's t-test was used to compare between sitagliptin treated and untreated cultures and the results were considered statistically significant when *P* values <0.05. No statistically significant difference between OD_600_ of the sitagliptin treated and untreated cultures after overnight incubation in LB broth (*P* = 0.0749).

To ensure absence of an effect of sitagliptin on growth, the optical densities of the bacterial suspension at 600 nm following overnight incubation in LB broth were measured in the presence or absence of sitagliptin (1 mg/ml). The experiment was done in triplicate and data are presented as the mean ± standard error. A *P* value < 0.05 was considered statistically significant using Student's t-test with (Graphpad Prism 5 software). No statistically significant difference was found between the turbidities of the bacterial suspension with or without sitagliptin indicating the lack of the effect of sitagliptin (sub MIC) on growth (Fig 1).

### Assessment of biofilm inhibition

**Fig 2:**
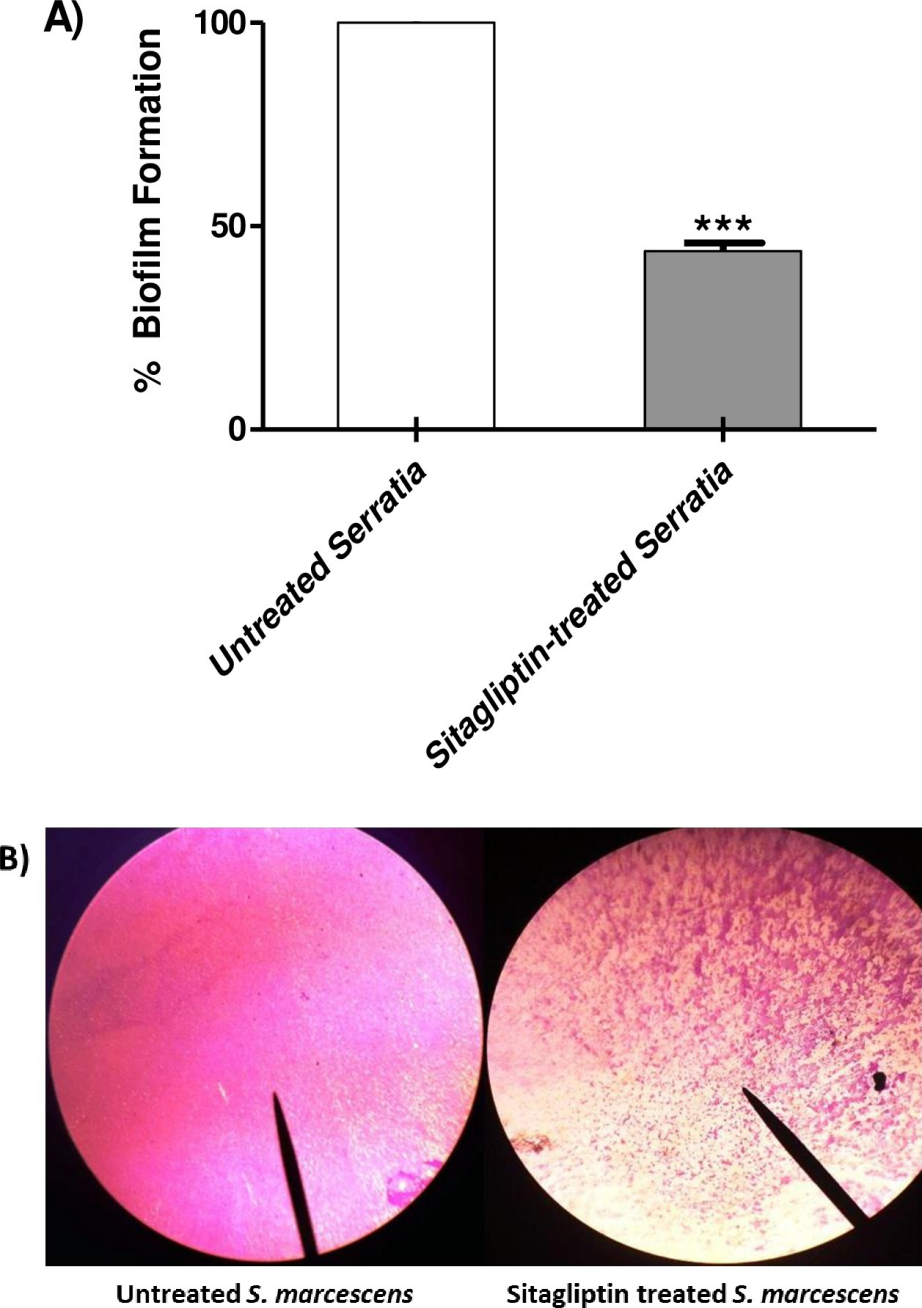
Biofilm inhibition of *S*. *marcescens* by sitagliptin. *S*. *marcescens* was cultured in presence or absence of sitagliptin (1mg/ml) in polystyrene micro-titer plate, and incubated at 37°C either for 24 h for evaluation of biofilm formation. A) Biofilm forming cells were stained by crystal violet, 33% glacial acetic acid was added to solubilize the dye and optical density was measured at 590 nm. Assays were performed in triplicate and reduction in biofilm formation was calculated. The absorbance of sitagliptin treated *S*. *marcescens* were expressed as mean ± standard error of percentage change from untreated *S*. *marcescens* control. The Student's t-test was used to compare between sitagliptin treated and untreated cultures and the results were considered statistically significant when *P* < 0.05. Sitagliptin at 1 mg/ml significant reduced the biofilm formation (*P* < 0.0001) and the percentage biofilm inhibition reached about 56%. B) The formed *S*. *marcescens* biofilm on glass slides in presence or absence of sitagliptin (1 mg/ml) was stained with crystal violet. The slides were examined under the light microscope, (left) untreated *S*. *marcescens* and (right) sitagliptin treated *S*. *marcescens* biofilms.

The biofilm formation was quantified to show the ability of sitagliptin to interfere with biofilm production. Significance of mean difference between sitagliptin treated and untreated bacteria was attested using Student's t-test on absolute values of optical density and the results were considered statistically significant when *P* < 0.05. Data were presented as mean ± standard error of percent change of biofilm formation by sitagliptin sub-MIC treated *S*. *marcescens* from untreated *S*. *marcescens* control. Sitagliptin at 1 mg/ml produced statistically significant reduction in biofilm biomass (*P <* 0.0001). The percentage biofilm inhibition reached about 56% (Fig 2A). To further explore the biofilm inhibiting activity of sitagliptin, light microscopic examination of biofilms formed on glass cover slips in the presence and absence of sitagliptin was performed. In the presence of sitagliptin, both the thickness and surface coverage were markedly decreased (Fig 2B).

### Inhibition of swimming and swarming

**Fig 3:**
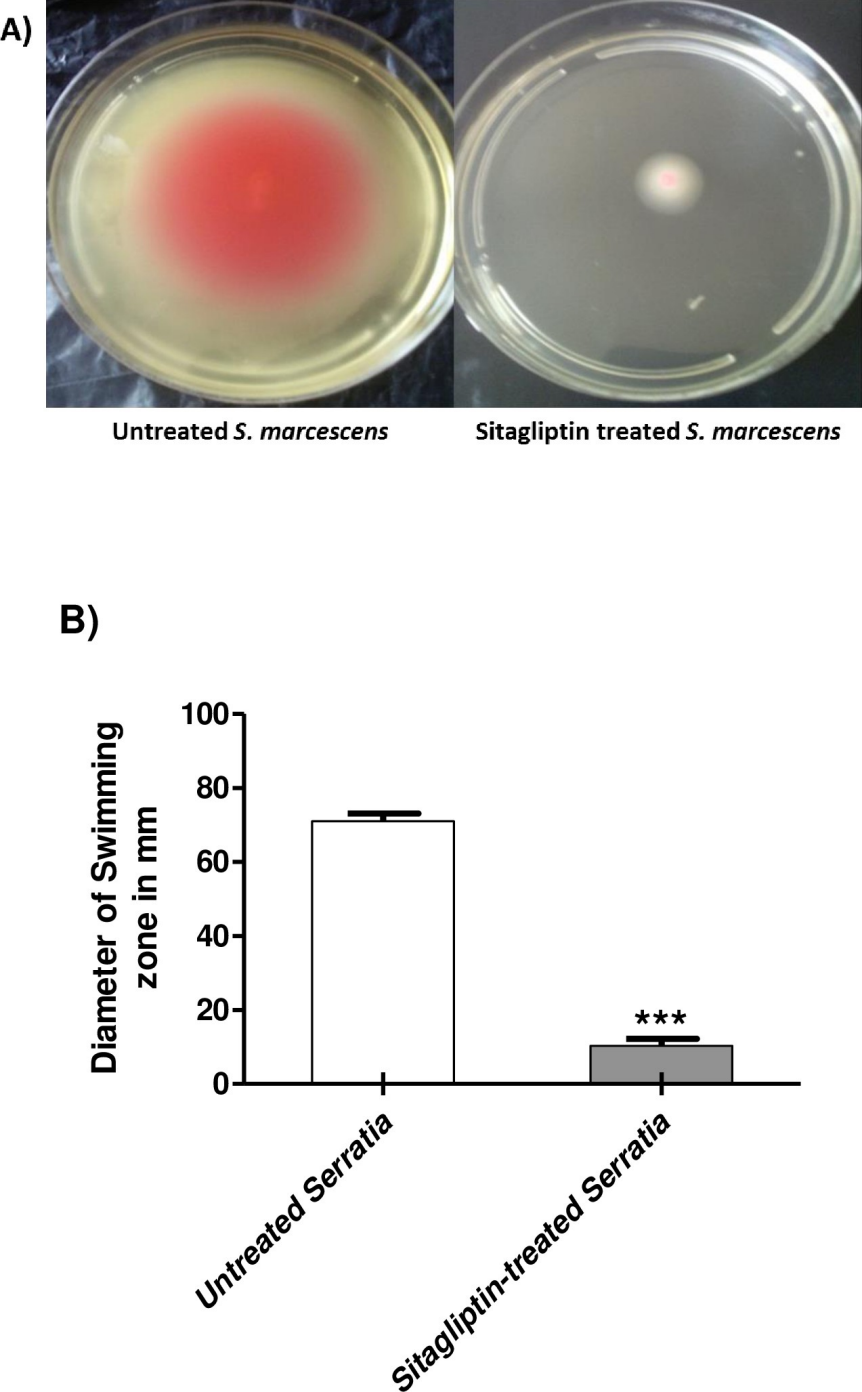
Inhibition of *S*. *marcescens* swimming motility by sitagliptin. LB agar plates with 0.3% agar with and without 1 mg/ml sitagliptin were prepared. The Student's t-test was used to compare between sitagliptin treated and untreated cultures and the results were considered statistically significant when *P* values < 0.05. A) 5μl from an overnight culture of *S*. *marcescens* (OD_600_ 0.4) was inoculated into the center of the plates. B) The zones of swimming were measured and the experiment was repeated in triplicates, sitagliptin significantly inhibited the swimming motility *S*. *marcescens* (*P* < 0.0001) and the percentage of reduction reached about 81%.

**Fig 4:**
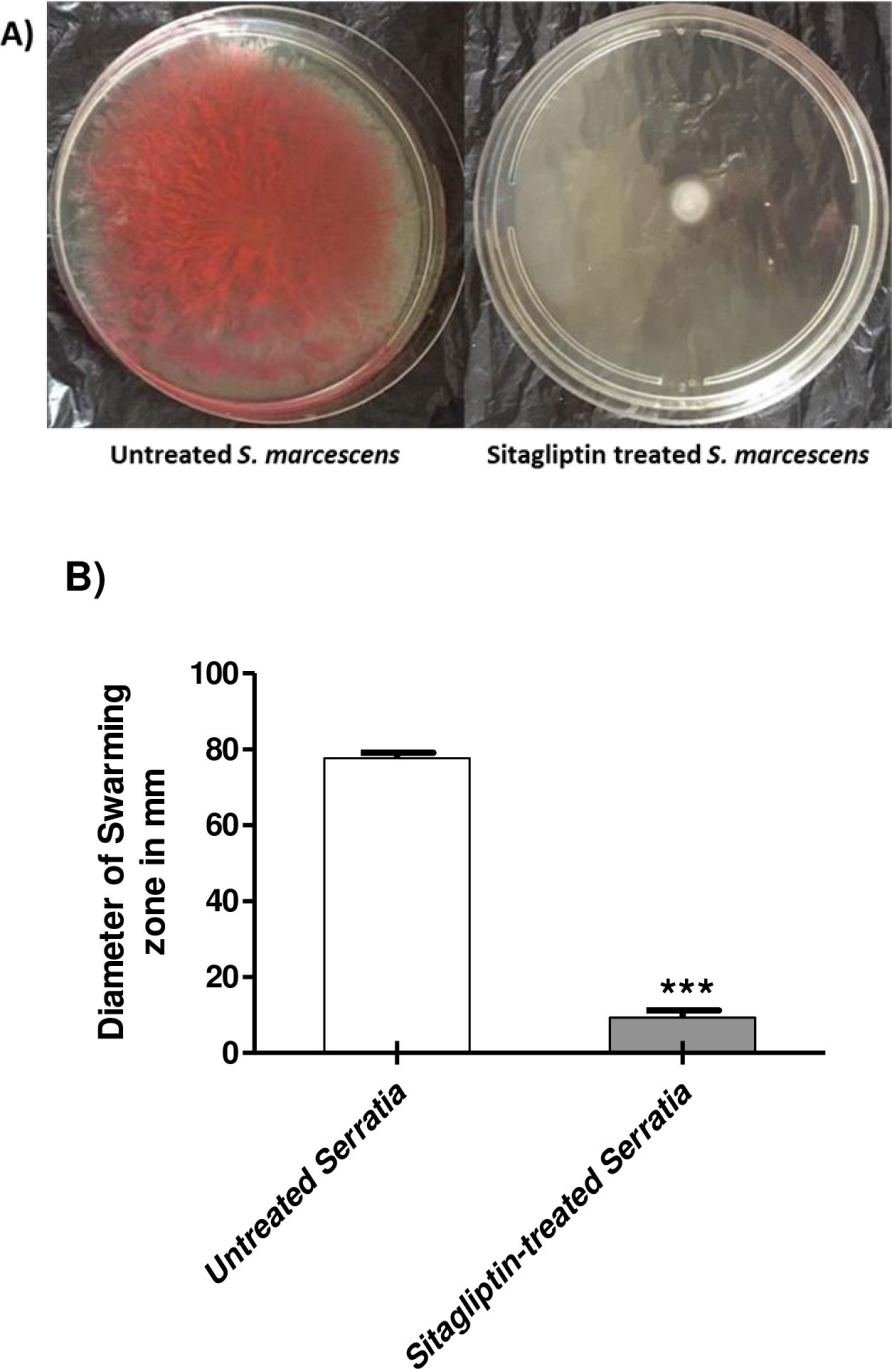
Inhibition of *S*. *marcescens* swarming motility by sitagliptin. LB agar plates with 0.5% agar with and without 1 mg/ml sitagliptin were prepared. The Student's t-test was used to compare between sitagliptin treated and untreated cultures and the results were considered statistically significant when *P* values < 0.05. A) 5μl from an overnight culture of *S*. *marcescens* (OD_600_ 0.4) was inoculated into the center of the plates. B) The zones of swarming were measured and the experiment was repeated in triplicates, sitagliptin significantly inhibited the swarming motility of *S*. *marcescens* (*P* < 0.0001) and the percentage of inhibition reached about 85%.

Swimming and swarming motility are important for adhesion and biofilm formation. The diameters of *S*. *marcescens* swimming and swarming agar were measured on 0.3% and 0.5% LB agar plates with and without 1 mg/ml sitagliptin. In the presence of sitagliptin, swimming motility was reduced by about 81%, while swarming motility was decreased by 85% (Figs 3 and 4 respectively). The experiments were repeated three times and the Student's t-test was used to compare between sitagliptin treated and untreated culture.

### Prodigiosin inhibition assay

**Fig 5:**
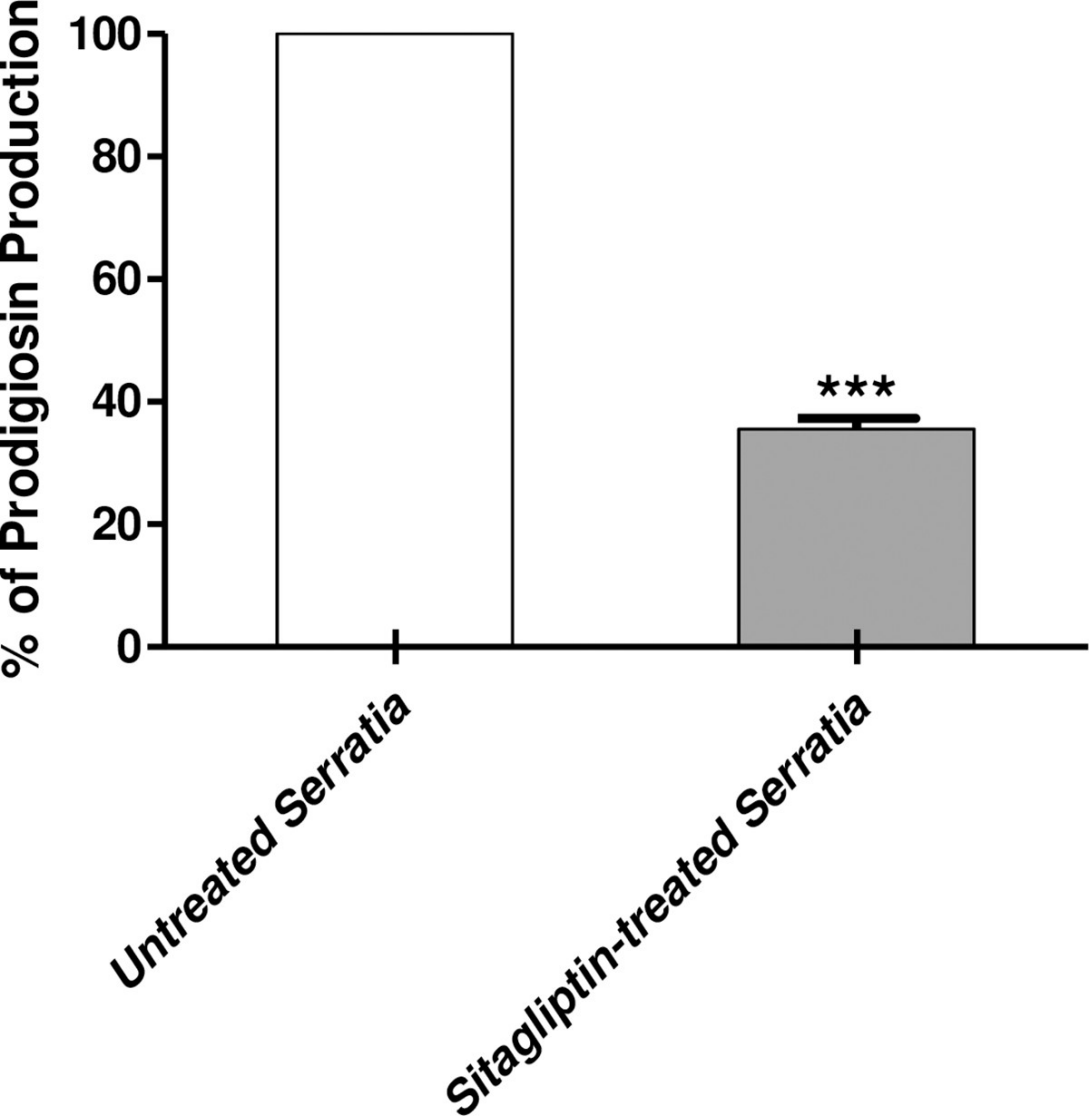
Inhibition of prodigiosin pigment of *S*. *marcescens* by sitagliptin. An optical density of overnight culture of *S*. *marcescens* was adjusted to 0.4 at 600nm, and inoculated in 2ml fresh LB broth at grown 28°C for 18 hr. The cells were collected and acidified to extract prodigiosin from cultures of *S*. *marcescens* in presence or absence of 1mg/ml sitagliptin. The growth of sitagliptin-treated and untreated cultures of *S*. *marcescens* was compared and the growth was not significantly affected by sitagliptin treatment. The experiment was made in triplicate, the absorbance was measured at 534 nm and the degree of inhibition was expressed as mean ± standard error of percentage change of prodigiosin production of sitagliptin treated *S*. *marcescens* from untreated *S*. *marcescens* control. Significantly, sitagliptin reduced the production of prodigiosin pigment of *S*. *marcescens* (*P* < 0.0001) with by about 65%.

Prodigiosin is a QS controlled pigment that is produced by *S*. *marcescens*. The absorbance of extracted prodigiosin from *S*. *marcescens* cultured in the presence and absence of sitagliptin was measured. The experiment was performed in triplicate and the Student's t-test was used to compare between absorbance values of sitagliptin sub-MIC treated and untreated *S*. *marcescens* and the results were considered statistically significant when *P* values < 0.05. The data obtained were presented as mean ± standard error of percentage change from untreated *S*. *marcescens* control (Fig 5). Sitagliptin showed a significant ability to inhibit prodigiosin production (*P* < 0.0001). The inhibition percentage achieved was 65%.

### Inhibition of protease production

**Fig 6:**
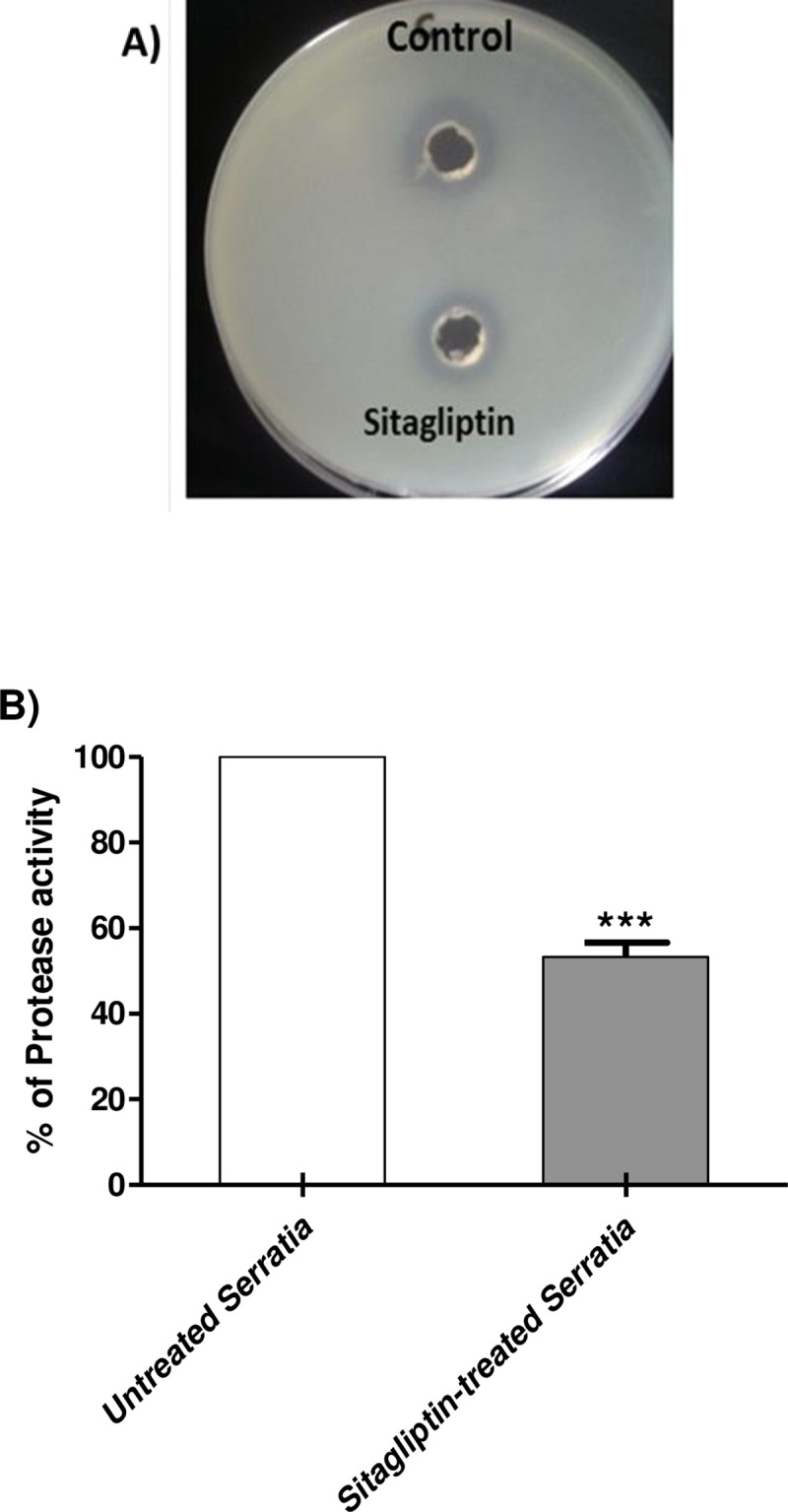
Inhibition of protease activity of *S*. *marcescens* by sitagliptin. A) *S*. *marcescens* overnight cultures in LB broth in the presence and absence of sitagliptin sub-MIC were adjusted to OD_600_ 0.4, centrifuged at 10,000 rpm for 15 min and the protease activities were measured by adding the supernatants in 100 μl aliquots to the wells made in skim milk agar plates (5%). The diameters of the clear zones surrounding the wells in skim milk agar plate were measured. B) The diameters of the clear zones surrounding the growth were measured. The experiment was done in triplicate and the degree of inhibition was determined. The Student's t-test was used to compare between sitagliptin treated and untreated *S*. *marcescens* and the results were considered statistically significant when *P* values < 0.05. Sitagliptin significantly decreased the protease activity of *S*. *marcescens* (*P* < 0.0001) with by 47%.

The skim milk agar method was used for the qualitative assay of protease inhibition. The experiment was done in triplicate and the degree of inhibition was determined. The clear zones obtained skim milk agar plates by protease produced in supernatants of sitagliptin sub-MIC treated and untreated *S*. *marcescens* cultures were measured in mm, significance was calculated using Student's t-test and the results were considered statistically significant when *P* values < 0.05. The results were expressed as mean ± standard error of protease production percent change by sitagliptin sub-MIC treated *S*. *marcescens* cultures from the protease obtained by untreated *S*. *marcescens* control on skim milk agar plates. Sitagliptin significantly interfered with the protease production (*P* < 0.0001) as shown by decreasing the clear zone of proteolysis by about 47% (Fig 6).

### Reduction of the expression of virulence genes of *S*. *marcescens* by sitagliptin

**Fig 7:**
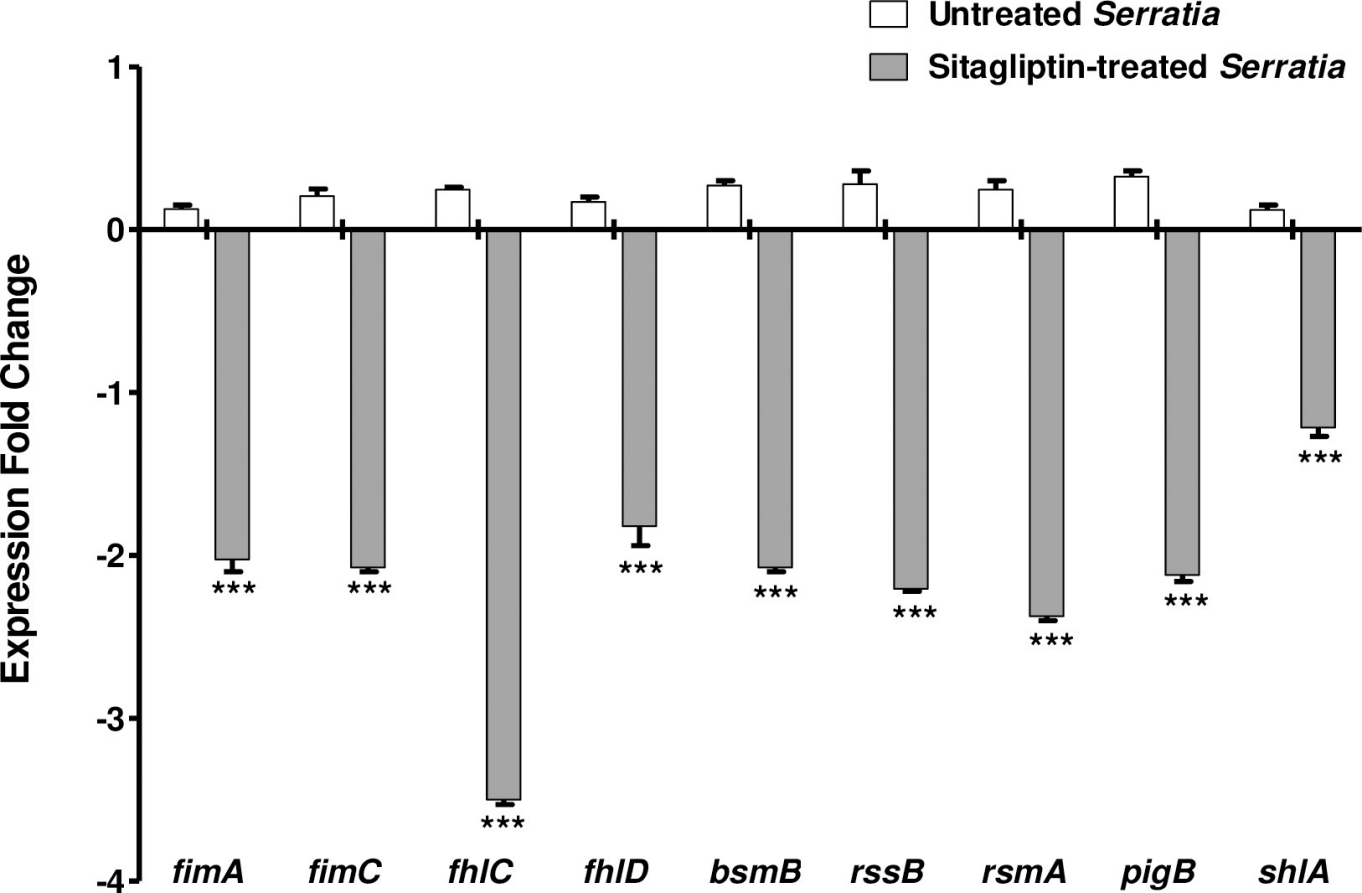
Sitagliptin downregulated virulence genes of *S*. *marcescens*. RNA was isolated from *S*. *marcescens* cultures treated and untreated with sitagliptin sub-MIC (OD_600_ 0.4) to be used in cDNA synthesis. The cDNA was amplified by qRT-PCR and changes in the expression of each gene were normalized in relation to the mean critical threshold values of housekeeping gene *rplU*. Expression fold change in gene expression in sitagliptin-treated *S*. *marcescens* was calculated by the 2^-ΔΔCT^ method and compared to untreated bacteria. The data shown are the mean ± standard errors from three experiments. *P<*0.05 was considered significant using Student’s t-test. Sitagliptin significantly decreased the expression of genes *fimA*, *fimC*, *flhC*, *flhD*, *bsmB*, *rssB*, *rsmA*, *pigP* and *shlA* (*P* < 0.0001).

To confirm the anti-virulence activity of sitagliptin, qRT-PCR was used (Fig 7). The expression of QS genes was evaluated in control *S*. *marcescens* and in sitagliptin-treated one by using 2^*-ΔΔCt*^ method. It is noteworthy to mention that the expression levels of all of of *fimA*, *fimC*, *flhC*, *flhD*, *bsmB*, *rssB*, *rsmA*, *pigP* and *shlA* genes were significantly reduced as compared to control untreated culture. The data shown are the mean ± standard error from three experiments, and *P* < 0.05 was considered significant using Student’s t-test. There was about 2-fold decrease in the expression levels of *fimA*, *fimC* and *bsmB* genes that encode and regulate fimbria upon treatment with sub-MIC of sitagliptin. The expression levels of swarming responsible genes were reduced in presence of sitaglibtin sub-MIC; 3- to 4-fold for *flhC* and 2- to3-fold for *flhD*, *rssB* and *rsmA*. In addition, sitaglibtin sub-MIC decreased the expression of the prodigiosin encoding gene *pigB* and the pore-forming toxin encoding gene *shlA* by 2-to 3-fold and less than 2-fold respectively.

### *In-vivo* protection activity of sitagliptin against *S*. *marcescens*

**Fig 8:**
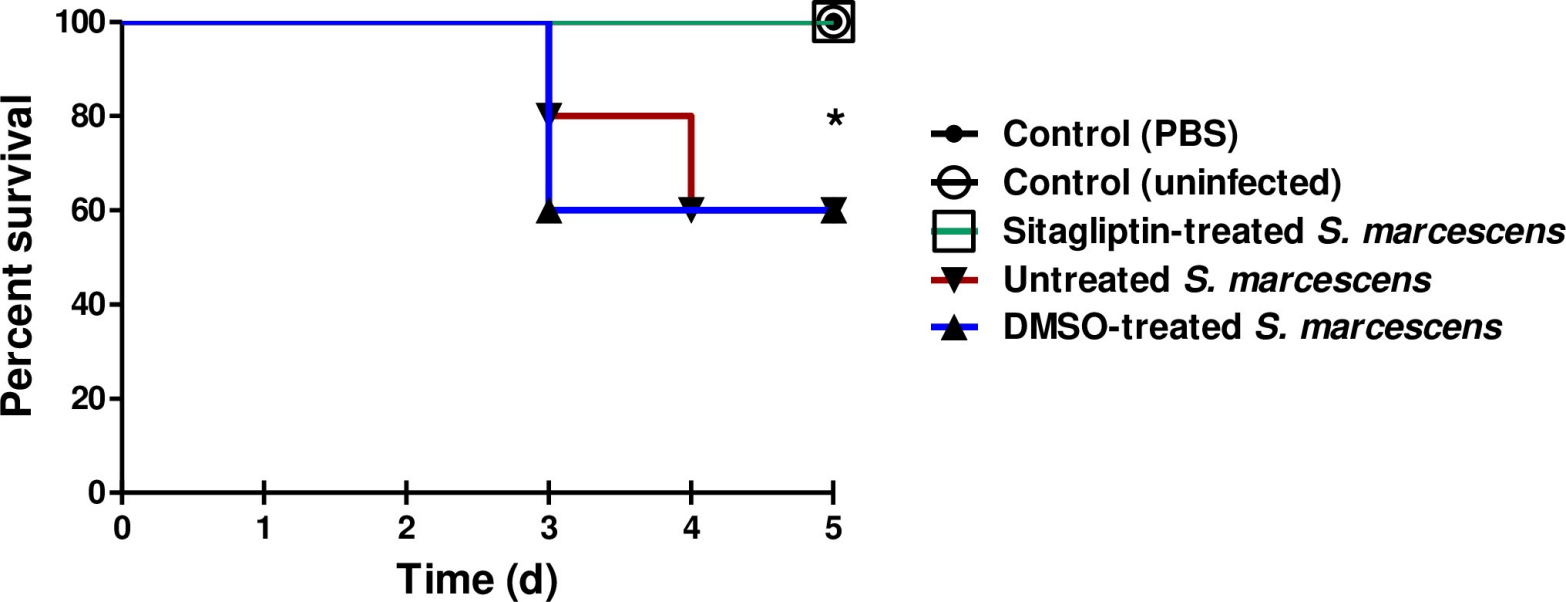
Sitagliptin protected mice from *S*. *marcescens*. Five mouse groups of healthy mice comprising 5 mice each were used. Group 1, mice were intraperitoneally injected with sitagliptin-treated *S*. *marcescens* in sterile PBS, group 2 was injected with DMSO-treated bacteria, group 3 was injected with untreated bacteria, group 4 was injected with sterile PBS and group 5 was left un-inoculated. Mice survival in each group was recorded every day over 5-days, plotted using Kaplan-Meier method and significance (*P* < 0.05) was calculated using Log-rank test, GraphPad Prism 5. All mice in groups 4 and 5 (negative control groups) survived, while only 60% of mice survived (3 out of 5 mice) in the groups comprising the DMSO-treated bacteria or untreated bacteria. In contrast to untreated *S*. marcescens, all mice injected with sitagliptin-treated *S*. *marcescens* survived, showing 100% survival, conferring 40% protection (Log rank test for trend *P* = 0.02).

The sitagliptin protection activity from *S*. *marcescens* virulence was further *in-vivo* evaluated. Five mouse groups of healthy female albino mice with the same weight, comprising 5 mice were used; the number of dead or alive animals in each group was recorded at the end of the experimental period. All mice in both negative control groups survived, while only 3 survived out of 5 (60% survival) in the mice injected with DMSO-treated bacteria or untreated bacteria. Interestingly, all mice injected with sitagliptin-treated *S*. *marcescens* showed 100% survival, conferring 40% protection in comparison to mice injected with un-treated *S*. *marcescens* (Fig 8). These findings clearly indicate that treatment of *S*. *marcescens* with sub-MIC of sitagliptin significantly reduced bacterial capacity to kill mice (*P* = 0.02) using Log rank test for trend (GraphPad Prism 5).

## Discussion

*S*. *marcescens* is Gram negative rod, with increasing clinical importance because of its frequent involvement in diverse nosocomial infections and considering it as one of opportunistic food-borne pathogens [2,4]. The pathogenesis of *S*. *marcescens* is owed greatly to several virulence factors such as its capability to produce extracellular enzymes, motility, resistance development to many antibiotics and others [2,5,6]. QS has gained much attention because it controls the physiological functions in *S*. *marcescens* and regulates swarming motility, sliding motility, hemolytic activity, production of biofilm, biosurfactant, antibiotics, lipase, protease, chitinase, nuclease and other activities [2]. QS modulates the bacterial virulence through regulation of the expression of virulence genes [12,13]. Targeting bacterial virulence controlled by QS is one approach that can be used to overcome the exaggerated bacterial pathogenesis and resistance to antibiotics [13,17]. In this direction, we and others were interested in exploring how to inhibit QS and bacterial virulence. Several compounds were screened for their anti-QS activity. Heterocyclic nitrogenous compounds were tested broadly as anti-QS inhibitors [18–20], particularly pyrazine derivatives that were successfully used to modulate QS in *Vibrio cholerae* [18] and triazole derivatives that showed efficient anti-QS activity [21–23]. Sitagliptin, a pyrazine derivative with triazole moiety, is a hypoglycaemic agent for type II diabetes patients via inhibition of dipeptidyl-peptidase IV [35]. In this study, augmented resistance of *S*. *marcescens* isolated from endotracheal aspiration of diabetic patient admitted to intensive care unit was found. The anti-diabetic drug sitagliptin was a member of the medication regimen which was introduced routinely to the patient. It was important to investigate if sitagliptin as a pyrazine-triazole derivative has any anti-QS and anti-virulence activities.

Sitagliptin was tested as anti-virulence agent against *S*. *marcescens* at sub MIC concentrations (0.1 MIC) which did not interfere with bacterial growth. Sitagliptin significantly inhibited the swarming and swimming motilities of *S*. *marcescens*; the inhibition percentages reached 81% and 85%, respectively. Swimming and swarming motilities are considerably important for adhesion and biofilm formation. Sitagliptin significantly inhibited biofilm formation with inhibition percentage of 56%. The production of extracellular enzymes by some bacterial strains has a crucial role in their virulence. *S*. *marcescens* has the capability to produce diverse enzymes as lipase, protease, chitinase, nuclease and others [2]. In this work, it was found that sitagliptin significantly inhibited the production of protease with inhibition percentage that reached 47%.

The red pigments prodiginine and its analogs prodigiosin, were first extracted from terrestrial bacterium *S*. *marcescens*. These pigments were widely used in wide array of biomedical and industrial applications including algicidal, bactericidal, anticancer, antiprotozoal, antimalarial, insecticidal, immunosuppressive agents and colorants [36]. Prodigiosin is a QS controlled pigment that is produced by *S*. *marcescens*. Sitagliptin showed a significant ability to inhibit prodigiosin production, with inhibition percentage of 65%.

After studying the effect of sitagliptin on the phenotypic behavior of *S*. *marcescens*, it was necessary to investigate the molecular basis of these inhibitions of QS activities through showing its effect on expression rates of some representative involved genes. Fimbria or pili are adhesive organelles of bacteria and are important in establishment of infections. *S*. *marcescens* pilus production has been reported and linked to biofilm formation [37]. Genetic analysis showed that the type I pili which encoded by the operon *fimABCD* are essential for *S*. *marcescens* biofilm formation in early stages [38]. Moreover, other transcriptional factors and proteins such as BsmA and BsmB are needed to increase the *S*. *marcescens* type I pilus production [32,38]. In this study, it was shown that sitagliptin significantly down regulated the expression of fimbrial A subunit protein FimB encoding genes *fimA* and *fimC* and type I fimbriae regulatory protein “BsmB-encoding” *bsmB* gene. These findings explain the significant reduction in biofilm formation in the presence of sitagliptin.

Swarming and biofilm formation are two important bacterial multicellular behaviors on surfaces. Bacteria can resist environmental stress by founding biofilm communities or survive by rapid spread to a better niche by swarming [39]. *S*. *marcescens* uses two-component system RssAB which comprises a sensor kinase and a specific cognate response regulator which suppresses master swarming regulator flhDC in early lag phase to control the surface migration timing without disturbing swarming development [40,41]. The flagellar regulatory master operon *flhDC*, temperature and nutrient status; all contribute to the regulation of swarming motility in *S*. *marcescens*. Moreover, RsmA is an essential component of the complex regulatory network that controls swarming [42,43]. The flagellar master regulator operon *flhDC*, composed of *flhC* and *flhD* genes encodes flagellar transcriptional regulator FlhC and flagellar transcriptional activator FlhD [44,45]. In this study, it was noted that sitagliptin significantly reduced the *S*. *marcescens* swimming and swarming motilities, which can be simply owed to the marked reduction in expression of genes which encode or regulate flagellar proteins *flhC*, *flhD*, *rssB* and *rsmA*.

The tripyrrole red QS controlled pigment, prodigiosin, is synthesized by *Serratia* through expression of the prodigiosin biosynthetic operon, pigA-N [46,47]. It was found that sitagliptin significantly reduced the expression of *pigB* gene. Multicellular organisms protect themselves from microbial invasion by different barriers. Single-layered epithelial cells of mucosae are the preferred tissue barriers that bacteria may invade. Pathogenic bacteria are equipped with arsenal of weapons to traverse these borders, while opportunistic pathogens, such as *S*. *marcescens*, can only cross epithelial barriers when tissues are destroyed or proliferate after physical trauma. Pore-forming toxins are potent virulence factors secreted by a large array of bacteria such as *S*. *marcescens* ShlA, which is secreted on host cell-to-cell junctions. ShlA is a member of a unique family of pore-forming toxins and is secreted by a two-component secretion system that uses Ca^2+^ influx triggering mechanism in the host cells [48,49]. Our results showed the significant capability of sitagliptin in reducing the expression of the pore-forming toxin encoding gene *shlA*.

From the previous results which showed a great compatibility between phenotypic observations and their genetic basis, sitagliptin was tested to investigate its protective effect against pathogenesis *in-vivo*. The protective activity of sitagliptin on *S*. *marcescens* pathogenesis was evaluated by the mice survival *in-vivo* model following the method of Kim *et al*. [34]. Interestingly, all the mice injected with sitagliptin (0.1 MIC) treated *S*. *marcescens* survived (100% survival, conferring 40% protection) as well as mice groups injected with sterile PBS or un-inoculated, in comparison to only 60% survival in mice group which was injected with untreated bacteria. Statistically, sitagliptin reduced the virulence of *S*. *marcescens*as shown by *in-vivo* experiment. This could be shown by the finding that mice injected with sitagliptin treated bacteria showed a trend for better survival compared to mice injected with DMSO treated bacteria or untreated bacteria. To this point, our *in-vitro* and *in-vivo* results declared that sitagliptin can serve as an efficient virulence inhibitor.

The most known QS gene regulatory systems in Gram negative bacteria are LuxI/LuxR systems which are controlled via direct binding of an autoinducer to a cytosolic transcription factor. The autoinducer synthase is the LuxI protein and the transcriptional regulator is the LuxR protein that links autoinducer as a ligand to dimerize and bind DNA to modulate QS-regulated genes [50,51]. LuxI/LuxR proteins are involved in QS signaling in *Pseudomonas aeruginosa*, *Vibrio* spp, *Erwinia carotovora*, *S*. *marcescens* and in wide variety of Gram negative bacteria [52]. Moreover, the N-acylhomoserine lactone (AHL) receptor SpnR protein is a member in LuxR family that modulates *S*. *marcescens* QS. SpnR binds to *S*. *marcescens* DNA on a lux-box like promoter to initiate a sequential QS process in the LuxR family [53]. In this context, it can be expected that the compounds which can regulate LuxI/LuxR system may have anti-QS activities, so then synthetic AHL analogues which inhibited Lux QS system were suggested as QS inhibitors [54]. Interestingly, Pyrazine dicarboxylic acid derivative were used successfully to regulate QS LuxO of Vibrio cholerae QS System [18]. The LuxO and its phosphorylated derivative LuxOP activate the transcription of the quorum regulatory RNAs (sRNAs) [55], which either positively or negatively post-transcriptionally regulate the expression of quorum sensing genes [56,57]. Compounds comprising the triazole moiety abundantly exists as antibacterial drugs such as macrolides, β-lactams and cephalosporin [19,23] and they showed anti-QS activities [23]. Triazole-containing natural AHL and its analogs strongly regulated the activity of LasR and AbaR [21]. Moreover, triazoles containing thymidine and isoxazole structures can serve as potential QS inhibitors [22]. The anti-QS activity of the compounds containing triazole moiety may be owed to their capability to bind diverse targets in the cell via dipole interactions and hydrogen bonding [19,20]. As a consequence, sitagliptin was suggested as a potential QS inhibitor due to its chemical structure that can be viewed as pyrazine derivative with triazole moiety. Interestingly, we showed in a previous study the promising *in-vitro* capability of sitagliptin in hindering the *Pseudomonas aeruginosa* virulence [58]. For all the above reasons, we were curious to go deeper and investigate both *in-vitro* and *in-vivo* anti-virulence activities of sitagliptin on different bacteria.

Finally, it can be concluded that QS is a well-known system that regulates biofilm, motility, extracellular toxins, enzymes and various virulence factors in pathogenic and opportunistic bacteria. Targeting bacterial virulence is less likely to induce the emergence of resistance because it exerts no pressure on bacterial growth. Instead, virulence inhibition attenuates bacteria and enhances the ability of immune system to eradicate them. Our studies provide an insight on the anti-virulence modulatory effect of sitagliptin on *S*. *marcescens*. Thus, sitagliptin could serve as a potent, target specific and non-toxic bacterial virulence inhibitor.

## References

[1] Su LH, Ou JT, Leu HS, Chiang PC, Chiu YP, et al (2003) Extended epidemic of nosocomial urinary tract infections caused by Serratia marcescens. J Clin Microbiol 41: 4726–4732. 10.1128/JCM.41.10.4726-4732.2003 14532211 PMC254321

[2] Van Houdt R, Givskov M, Michiels CW (2007) Quorum sensing in Serratia. FEMS Microbiol Rev 31: 407–424. 10.1111/j.1574-6976.2007.00071.x 17459113

[3] Jones RN (2010) Microbial etiologies of hospital-acquired bacterial pneumonia and ventilator-associated bacterial pneumonia. Clin Infect Dis 51 Suppl 1: S81–87. 20597676 10.1086/653053

[4] Cristina ML, Sartini M, Spagnolo AM (2019) Serratia marcescens Infections in Neonatal Intensive Care Units (NICUs). Int J Environ Res Public Health 16. 10.3390/ijerph16040610 PMC6406414 30791509

[5] Stock I, Burak S, Sherwood KJ, Gruger T, Wiedemann B (2003) Natural antimicrobial susceptibilities of strains of 'unusual' Serratia species: S. ficaria, S. fonticola, S. odorifera, S. plymuthica and S. rubidaea. J Antimicrob Chemother 51: 865–885. 10.1093/jac/dkg156 12654765

[6] Traub WH (2000) Antibiotic susceptibility of Serratia marcescens and Serratia liquefaciens. Chemotherapy 46: 315–321. 10.1159/000007304 10965096

[7] Davies DG, Parsek MR, Pearson JP, Iglewski BH, Costerton JW, et al (1998) The involvement of cell-to-cell signals in the development of a bacterial biofilm. Science 280: 295–298. 10.1126/science.280.5361.295 9535661

[8] Coulthurst SJ, Williamson NR, Harris AK, Spring DR, Salmond GP (2006) Metabolic and regulatory engineering of Serratia marcescens: mimicking phage-mediated horizontal acquisition of antibiotic biosynthesis and quorum-sensing capacities. Microbiology 152: 1899–1911. 10.1099/mic.0.28803-0 16804166

[9] Horng YT, Deng SC, Daykin M, Soo PC, Wei JR, et al (2002) The LuxR family protein SpnR functions as a negative regulator of N-acylhomoserine lactone-dependent quorum sensing in Serratia marcescens. Mol Microbiol 45: 1655–1671. 10.1046/j.1365-2958.2002.03117.x 12354232

[10] Thomson NR, Crow MA, McGowan SJ, Cox A, Salmond GP (2000) Biosynthesis of carbapenem antibiotic and prodigiosin pigment in Serratia is under quorum sensing control. Mol Microbiol 36: 539–556. 10.1046/j.1365-2958.2000.01872.x 10844645

[11] Van Houdt R, Moons P, Aertsen A, Jansen A, Vanoirbeek K, et al (2007) Characterization of a luxI/luxR-type quorum sensing system and N-acyl-homoserine lactone-dependent regulation of exo-enzyme and antibacterial component production in Serratia plymuthica RVH1. Res Microbiol 158: 150–158. 10.1016/j.resmic.2006.11.008 17258895

[12] Mion S, Remy B, Plener L, Bregeon F, Chabriere E, et al (2019) Quorum Quenching Lactonase Strengthens Bacteriophage and Antibiotic Arsenal Against Pseudomonas aeruginosa Clinical Isolates. Front Microbiol 10: 2049 10.3389/fmicb.2019.02049 31551983 PMC6734170

[13] Gebreyohannes G, Nyerere A, Bii C, Sbhatu DB (2019) Challenges of intervention, treatment, and antibiotic resistance of biofilm-forming microorganisms. Heliyon 5: e02192 10.1016/j.heliyon.2019.e02192 31463386 PMC6709409

[14] Pushpakom S, Iorio F, Eyers PA, Escott KJ, Hopper S, et al (2019) Drug repurposing: progress, challenges and recommendations. Nat Rev Drug Discov 18: 41–58. 10.1038/nrd.2018.168 30310233

[15] Adonizio A, Kong KF, Mathee K (2008) Inhibition of quorum sensing-controlled virulence factor production in Pseudomonas aeruginosa by South Florida plant extracts. Antimicrob Agents Chemother 52: 198–203. 10.1128/AAC.00612-07 17938186 PMC2223872

[16] Kalia VC, Purohit HJ (2011) Quenching the quorum sensing system: potential antibacterial drug targets. Crit Rev Microbiol 37: 121–140. 10.3109/1040841X.2010.532479 21271798

[17] Memariani H, Memariani M, Ghasemian A (2019) An overview on anti-biofilm properties of quercetin against bacterial pathogens. World J Microbiol Biotechnol 35: 143 10.1007/s11274-019-2719-5 31493142

[18] Hema M, Vasudevan S, Balamurugan P, Adline Princy S (2017) Modulating the Global Response Regulator, LuxO of V. cholerae Quorum Sensing System Using a Pyrazine Dicarboxylic Acid Derivative (PDCA(py)): An Antivirulence Approach. Front Cell Infect Microbiol 7: 441 10.3389/fcimb.2017.00441 29075619 PMC5643417

[19] Senwar KR, Sharma P, Reddy TS, Jeengar MK, Nayak VL, et al (2015) Spirooxindole-derived morpholine-fused-1,2,3-triazoles: Design, synthesis, cytotoxicity and apoptosis inducing studies. Eur J Med Chem 102: 413–424. 10.1016/j.ejmech.2015.08.017 26301558

[20] Fu O, Pukin AV, Quarles van Ufford HC, Kemmink J, de Mol NJ, et al (2015) Functionalization of a Rigid Divalent Ligand for LecA, a Bacterial Adhesion Lectin. ChemistryOpen 4: 463–470. 10.1002/open.201402171 26478841 PMC4603407

[21] Hansen MR, Jakobsen TH, Bang CG, Cohrt AE, Hansen CL, et al (2015) Triazole-containing N-acyl homoserine lactones targeting the quorum sensing system in Pseudomonas aeruginosa. Bioorg Med Chem 23: 1638–1650. 10.1016/j.bmc.2015.01.038 25716005

[22] Zhang DW, Zhang YM, Li J, Zhao TQ, Gu Q, et al (2017) Ultrasonic-assisted synthesis of 1,4-disubstituted 1,2,3-triazoles via various terminal acetylenes and azide and their quorum sensing inhibition. Ultrason Sonochem 36: 343–353. 10.1016/j.ultsonch.2016.12.011 28069219

[23] Zhang B, Guo B, Bai Y, Lu H, Dong Y (2018) Synthesis and Biological Evaluation of Azamacrolide Comprising the Triazole Moiety as Quorum Sensing Inhibitors. Molecules 23. 10.3390/molecules23051086 PMC6102594 29734673

[24] Abbas HA, Hegazy WAH (2017) Targeting the virulence factors of Serratia marcescens by ambroxol. Roumanian Archives of Microbiology and Immunology 76: 27–32.

[25] Nalca Y, Jansch L, Bredenbruch F, Geffers R, Buer J, et al (2006) Quorum-sensing antagonistic activities of azithromycin in Pseudomonas aeruginosa PAO1: a global approach. Antimicrob Agents Chemother 50: 1680–1688. 10.1128/AAC.50.5.1680-1688.2006 16641435 PMC1472232

[26] Choo JH, Rukayadi Y, Hwang JK (2006) Inhibition of bacterial quorum sensing by vanilla extract. Lett Appl Microbiol 42: 637–641. 10.1111/j.1472-765X.2006.01928.x 16706905

[27] Issac Abraham SV, Palani A, Ramaswamy BR, Shunmugiah KP, Arumugam VR (2011) Antiquorum sensing and antibiofilm potential of Capparis spinosa. Arch Med Res 42: 658–668. 10.1016/j.arcmed.2011.12.002 22222491

[28] Sarkar R, Chaudhary SK, Sharma A, Yadav KK, Nema NK, et al (2014) Anti-biofilm activity of Marula—a study with the standardized bark extract. J Ethnopharmacol 154: 170–175. 10.1016/j.jep.2014.03.067 24742751

[29] Slater H, Crow M, Everson L, Salmond GP (2003) Phosphate availability regulates biosynthesis of two antibiotics, prodigiosin and carbapenem, in Serratia via both quorum-sensing-dependent and -independent pathways. Mol Microbiol 47: 303–320. 10.1046/j.1365-2958.2003.03295.x 12519208

[30] Matsuyama T, Kaneda K, Nakagawa Y, Isa K, Hara-Hotta H, et al (1992) A novel extracellular cyclic lipopeptide which promotes flagellum-dependent and -independent spreading growth of Serratia marcescens. J Bacteriol 174: 1769–1776. 10.1128/jb.174.6.1769-1776.1992 1548227 PMC205777

[31] Salini R, Pandian SK (2015) Interference of quorum sensing in urinary pathogen Serratia marcescens by Anethum graveolens. Pathog Dis 73: ftv038 10.1093/femspd/ftv038 26013821

[32] Ramanathan S, Ravindran D, Arunachalam K, Arumugam VR (2018) Inhibition of quorum sensing-dependent biofilm and virulence genes expression in environmental pathogen Serratia marcescens by petroselinic acid. Antonie Van Leeuwenhoek 111: 501–515. 10.1007/s10482-017-0971-y 29101490

[33] Srinivasan R, Mohankumar R, Kannappan A, Karthick Raja V, Archunan G, et al (2017) Exploring the Anti-quorum Sensing and Antibiofilm Efficacy of Phytol against Serratia marcescens Associated Acute Pyelonephritis Infection in Wistar Rats. Front Cell Infect Microbiol 7: 498 10.3389/fcimb.2017.00498 29259923 PMC5723315

[34] Kim HS, Lee SH, Byun Y, Park HD (2015) 6-Gingerol reduces Pseudomonas aeruginosa biofilm formation and virulence via quorum sensing inhibition. Sci Rep 5: 8656 10.1038/srep08656 25728862 PMC4345325

[35] Ahren B (2019) DPP-4 Inhibition and the Path to Clinical Proof. Front Endocrinol (Lausanne) 10: 376. 31275243 10.3389/fendo.2019.00376 PMC6593050

[36] Sakai-Kawada FE, Ip CG, Hagiwara KA, Awaya JD (2019) Biosynthesis and Bioactivity of Prodiginine Analogs in Marine Bacteria, Pseudoalteromonas: A Mini Review. Front Microbiol 10: 1715 10.3389/fmicb.2019.01715 31396200 PMC6667630

[37] Shanks RM, Stella NA, Brothers KM, Polaski DM (2016) Exploitation of a "hockey-puck" phenotype to identify pilus and biofilm regulators in Serratia marcescens through genetic analysis. Can J Microbiol 62: 83–93. 10.1139/cjm-2015-0566 26640000 PMC4739789

[38] Shanks RM, Stella NA, Kalivoda EJ, Doe MR, O'Dee DM, et al (2007) A Serratia marcescens OxyR homolog mediates surface attachment and biofilm formation. J Bacteriol 189: 7262–7272. 10.1128/JB.00859-07 17675374 PMC2168423

[39] Tsai YH, Wei JR, Lin CS, Chen PH, Huang S, et al (2011) RssAB signaling coordinates early development of surface multicellularity in Serratia marcescens. PLoS One 6: e24154 10.1371/journal.pone.0024154 21887380 PMC3162612

[40] Wei CF, Tsai YH, Tsai SH, Lin CS, Chang CJ, et al (2017) Cross-talk between bacterial two-component systems drives stepwise regulation of flagellar biosynthesis in swarming development. Biochem Biophys Res Commun 489: 70–75. 10.1016/j.bbrc.2017.05.077 28522292

[41] Lin CS, Tsai YH, Chang CJ, Tseng SF, Wu TR, et al (2016) An iron detection system determines bacterial swarming initiation and biofilm formation. Sci Rep 6: 36747 10.1038/srep36747 27845335 PMC5109203

[42] Ang S, Horng YT, Shu JC, Soo PC, Liu JH, et al (2001) The role of RsmA in the regulation of swarming motility in Serratia marcescens. J Biomed Sci 8: 160–169. 10.1007/bf02256408 11287746

[43] Liaw SJ, Lai HC, Ho SW, Luh KT, Wang WB (2003) Role of RsmA in the regulation of swarming motility and virulence factor expression in Proteus mirabilis. J Med Microbiol 52: 19–28. 10.1099/jmm.0.05024-0 12488561

[44] Iguchi A, Nagaya Y, Pradel E, Ooka T, Ogura Y, et al (2014) Genome evolution and plasticity of Serratia marcescens, an important multidrug-resistant nosocomial pathogen. Genome Biol Evol 6: 2096–2110. 10.1093/gbe/evu160 25070509 PMC4231636

[45] Nicholson WL, Leonard MT, Fajardo-Cavazos P, Panayotova N, Farmerie WG, et al (2013) Complete Genome Sequence of Serratia liquefaciens Strain ATCC 27592. Genome Announc 1. 10.1128/genomeA.00548-13 PMC3744671 23950115

[46] Brothers KM, Stella NA, Romanowski EG, Kowalski RP, Shanks RM (2015) EepR Mediates Secreted-Protein Production, Desiccation Survival, and Proliferation in a Corneal Infection Model. Infect Immun 83: 4373–4382. 10.1128/IAI.00466-15 26324535 PMC4598396

[47] Stella NA, Fender JE, Lahr RM, Kalivoda EJ, Shanks RM (2012) The LysR Transcription Factor, HexS, Is Required for Glucose Inhibition of Prodigiosin Production by Serratia marcescens. Adv Microbiol 2. 10.4236/aim.2012.24065 PMC3865871 24358451

[48] Reboud E, Bouillot S, Patot S, Beganton B, Attree I, et al (2017) Pseudomonas aeruginosa ExlA and Serratia marcescens ShlA trigger cadherin cleavage by promoting calcium influx and ADAM10 activation. PLoS Pathog 13: e1006579 10.1371/journal.ppat.1006579 28832671 PMC5584975

[49] Lazzaro M, Krapf D, Garcia Vescovi E (2019) Selective blockage of Serratia marcescens ShlA by nickel inhibits the pore-forming toxin-mediated phenotypes in eukaryotic cells. Cell Microbiol 21: e13045 10.1111/cmi.13045 31099073

[50] Engebrecht J, Silverman M (1984) Identification of genes and gene products necessary for bacterial bioluminescence. Proc Natl Acad Sci U S A 81: 4154–4158. 10.1073/pnas.81.13.4154 6377310 PMC345387

[51] Fuqua WC, Winans SC, Greenberg EP (1994) Quorum sensing in bacteria: the LuxR-LuxI family of cell density-responsive transcriptional regulators. J Bacteriol 176: 269–275. 10.1128/jb.176.2.269-275.1994 8288518 PMC205046

[52] Swift S, Winson MK, Chan PF, Bainton NJ, Birdsall M, et al (1993) A novel strategy for the isolation of luxI homologues: evidence for the widespread distribution of a LuxR:LuxI superfamily in enteric bacteria. Mol Microbiol 10: 511–520. 10.1111/j.1365-2958.1993.tb00923.x 7968529

[53] Takayama Y, Kato N (2016) In vitro analysis of essential binding sites on the promoter of the Serratia marcescens spn operon with the quorum-sensing receptor SpnR. Biotechnol Bioeng 113: 2513–2517. 10.1002/bit.26013 27217017

[54] Wang W, Morohoshi T, Ikeda T, Chen L (2008) Inhibition of Lux quorum-sensing system by synthetic N-acyl-L-homoserine lactone analogous. Acta Biochim Biophys Sin (Shanghai) 40: 1023–1028. 19089300 10.1111/j.1745-7270.2008.00490.x

[55] Tu KC, Bassler BL (2007) Multiple small RNAs act additively to integrate sensory information and control quorum sensing in Vibrio harveyi. Genes Dev 21: 221–233. 10.1101/gad.1502407 17234887 PMC1770904

[56] Feng L, Rutherford ST, Papenfort K, Bagert JD, van Kessel JC, et al (2015) A qrr noncoding RNA deploys four different regulatory mechanisms to optimize quorum-sensing dynamics. Cell 160: 228–240. 10.1016/j.cell.2014.11.051 25579683 PMC4313533

[57] Ball AS, Chaparian RR, van Kessel JC (2017) Quorum Sensing Gene Regulation by LuxR/HapR Master Regulators in Vibrios. J Bacteriol 199. 10.1128/JB.00105-17 PMC5585708 28484045

[58] Abbas HA, Shaldam MA, Eldamasi D (2020) Curtailing Quorum Sensing in Pseudomonas aeruginosa by Sitagliptin. Curr Microbiol. 10.1007/s00284-020-01909-4 32020464

